# Supplementary material for: TurboID reveals the proxiomes of Chlamydomonas proteins involved in thylakoid biogenesis and stress response
Source: Plant Physiol. 2023 Jun 13;193(3):1772–96. doi: 10.1093/plphys/kiad335 (PMC10602608; doi:10.1093/plphys/kiad335)
Supplement: kiad335_Supplementary_Data [file kiad335_supplementary_data.zip › Original images used for Supplemental Figure S5C.pptx]

## Slide 1
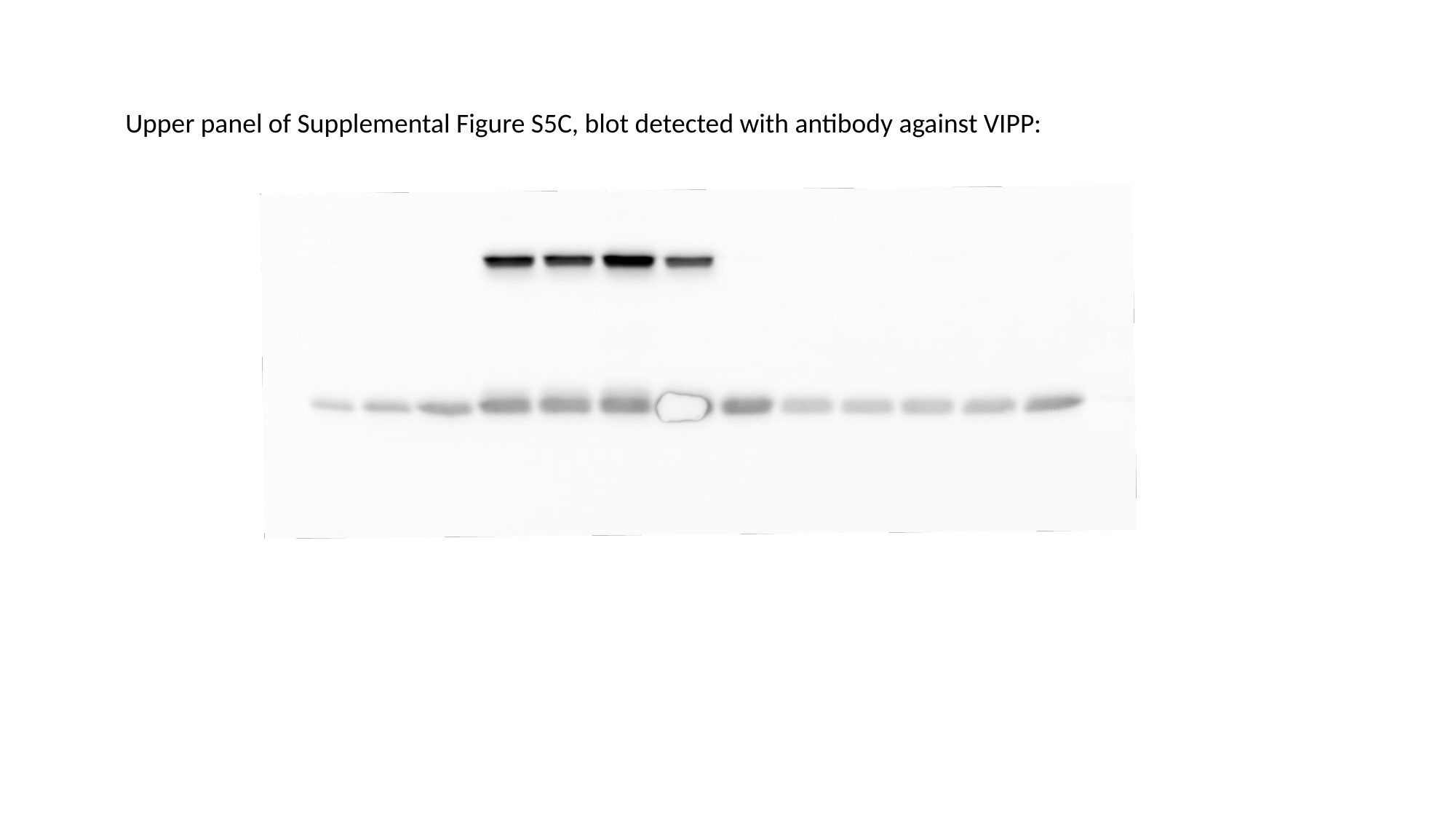

Upper panel of Supplemental Figure S5C, blot detected with antibody against VIPP:

## Slide 2
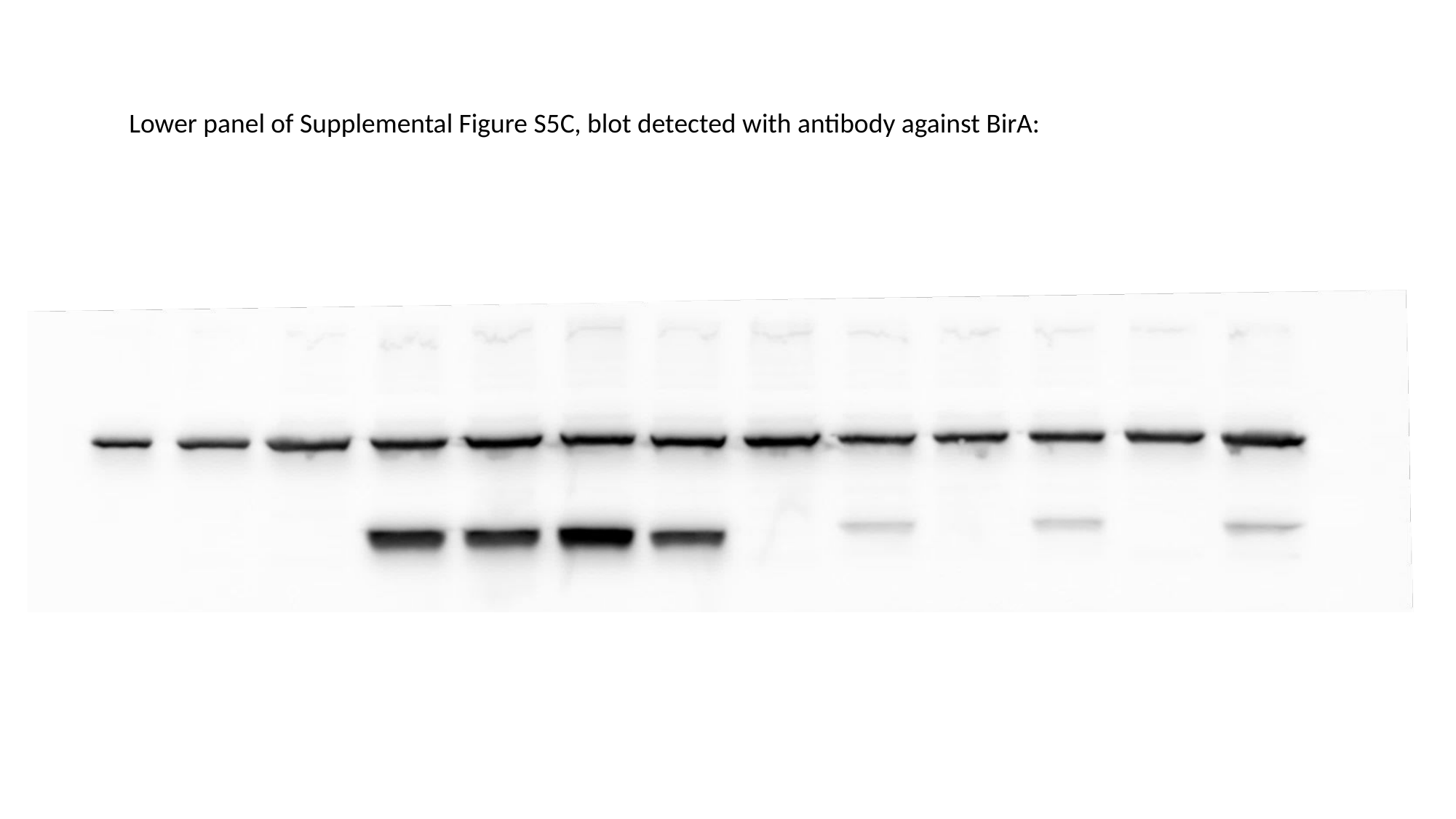

Lower panel of Supplemental Figure S5C, blot detected with antibody against BirA:
