# Supplementary material for: TurboID reveals the proxiomes of Chlamydomonas proteins involved in thylakoid biogenesis and stress response
Source: Plant Physiol. 2023 Jun 13;193(3):1772–96. doi: 10.1093/plphys/kiad335 (PMC10602608; doi:10.1093/plphys/kiad335)
Supplement: kiad335_Supplementary_Data [file kiad335_supplementary_data.zip › PP2023RA00327R1_Supplemental_Material.pdf]

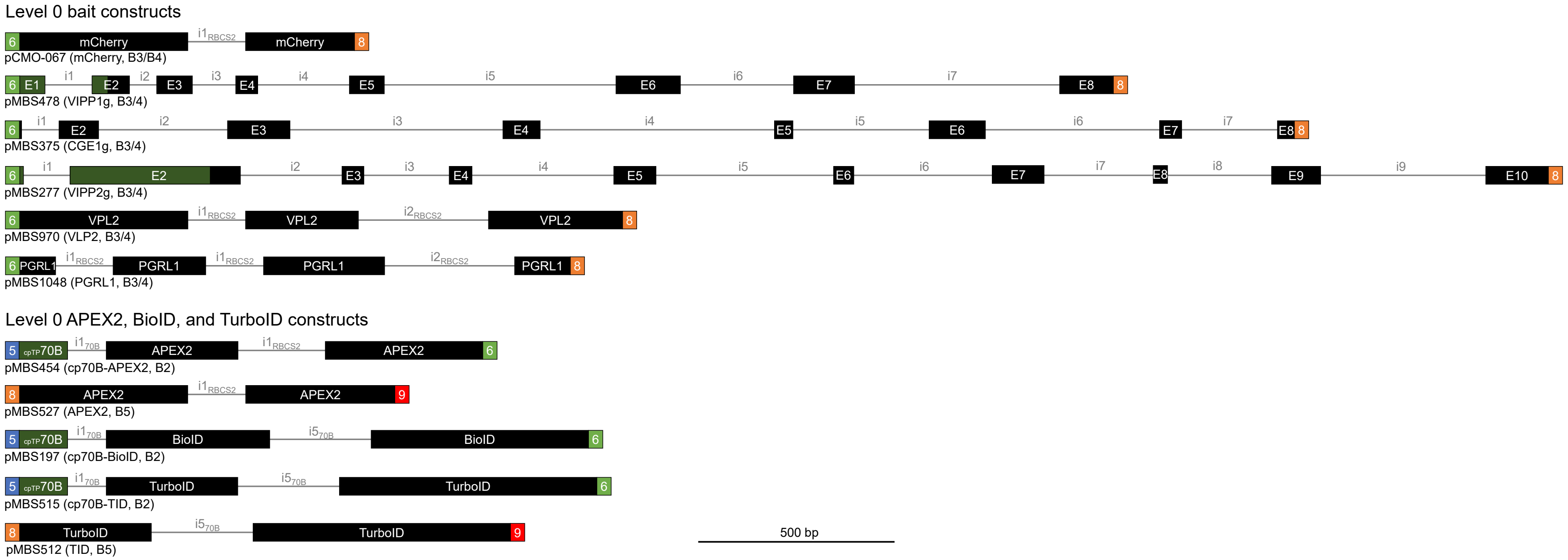

Supplemental Figure S1. Level 0 constructs for baits and biotin activators used in this study.
Level 0 constructs for coding sequences (CDS) of baits (top) and APEX2, BioID, and TurboID (bottom). Fusion sites according to Patron et al. (2015) are indicated as colored boxes using the color code from Crozet et al. (2018). Exons are shown as black boxes. Exons from native genes are designated as “E”, artificial exons generated by the insertion of introns into codon-optimized CDS are indicated by the name of the CDS. cp70B is the chloroplast transit peptide of HSP70B. Introns are shown as grey lines and the source of each intron is indicated on top (“i” and a number denotes the native introns). All constructs are drawn to scale. Supports Figures 1, 2, and 5.

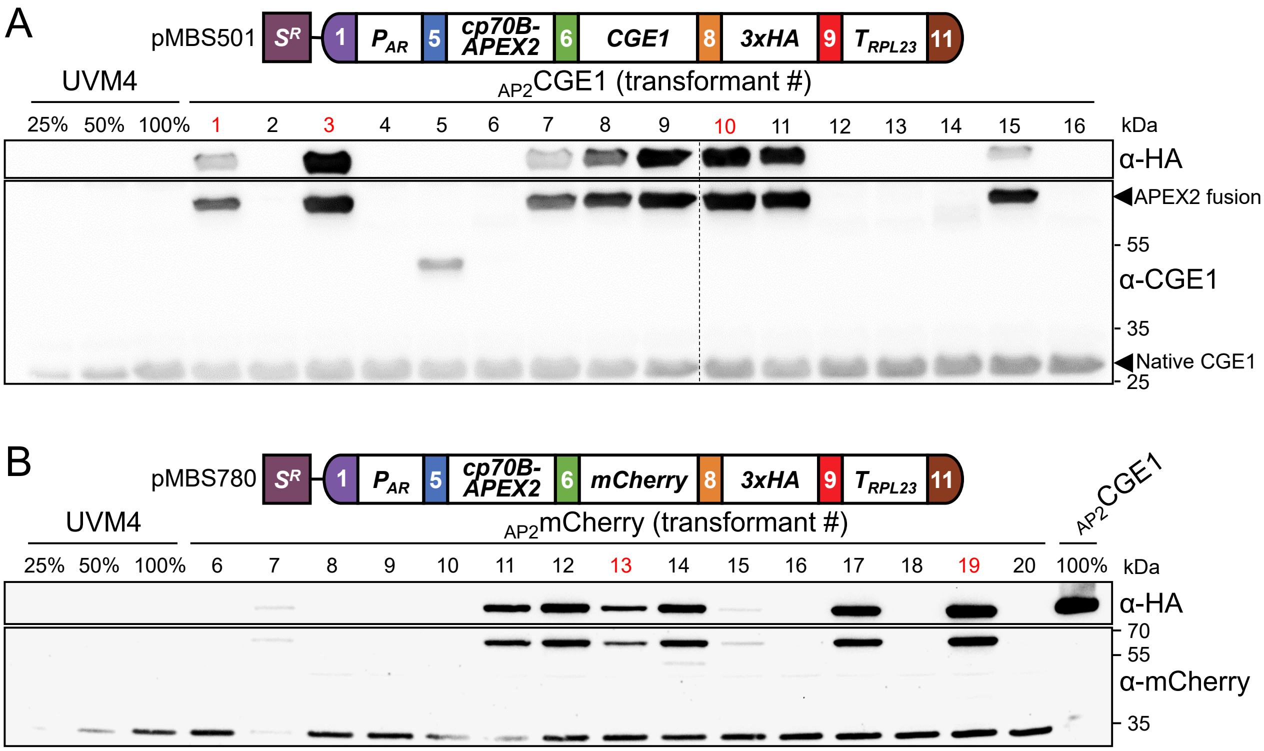

Supplemental Figure S2. Screening for transformants accumulating CGE1 and mCherry with N-terminal fusions to APEX2.

(A) Level 2 construct pMBS501 conferring resistance to spectinomycin (S^R) and driving the production of CGE1 fused N-terminally to APEX2 ($_{AP2}$CGE1). The fusion protein is targeted to the chloroplast via the HSP70B chloroplast transit peptide (cp70B). Total cell protein extracts corresponding to 2 µg chlorophyll for each transformant generated with pMBS501 were separated by SDS-PAGE and analyzed by immunoblotting using an antibody against the HA epitope and against CGE1. The recipient strain UVM4 served as negative control. The transformant number is given on top of each panel and transformants in red were chosen for further analysis. The positions of native CGE1 and CGE1 fused to APEX2 are indicated.

(B) Level 2 construct pMBS780 for the production of chloroplast targeted mCherry fused N-terminally to APEX2 ($_{AP2}$mCherry). The analysis of transformants was done as in (A) but using antibodies against the HA tag and against mCherry. Protein extract from $_{AP2}$CGE1 transformant #3 was used as positive control.

Supports Figure 1.

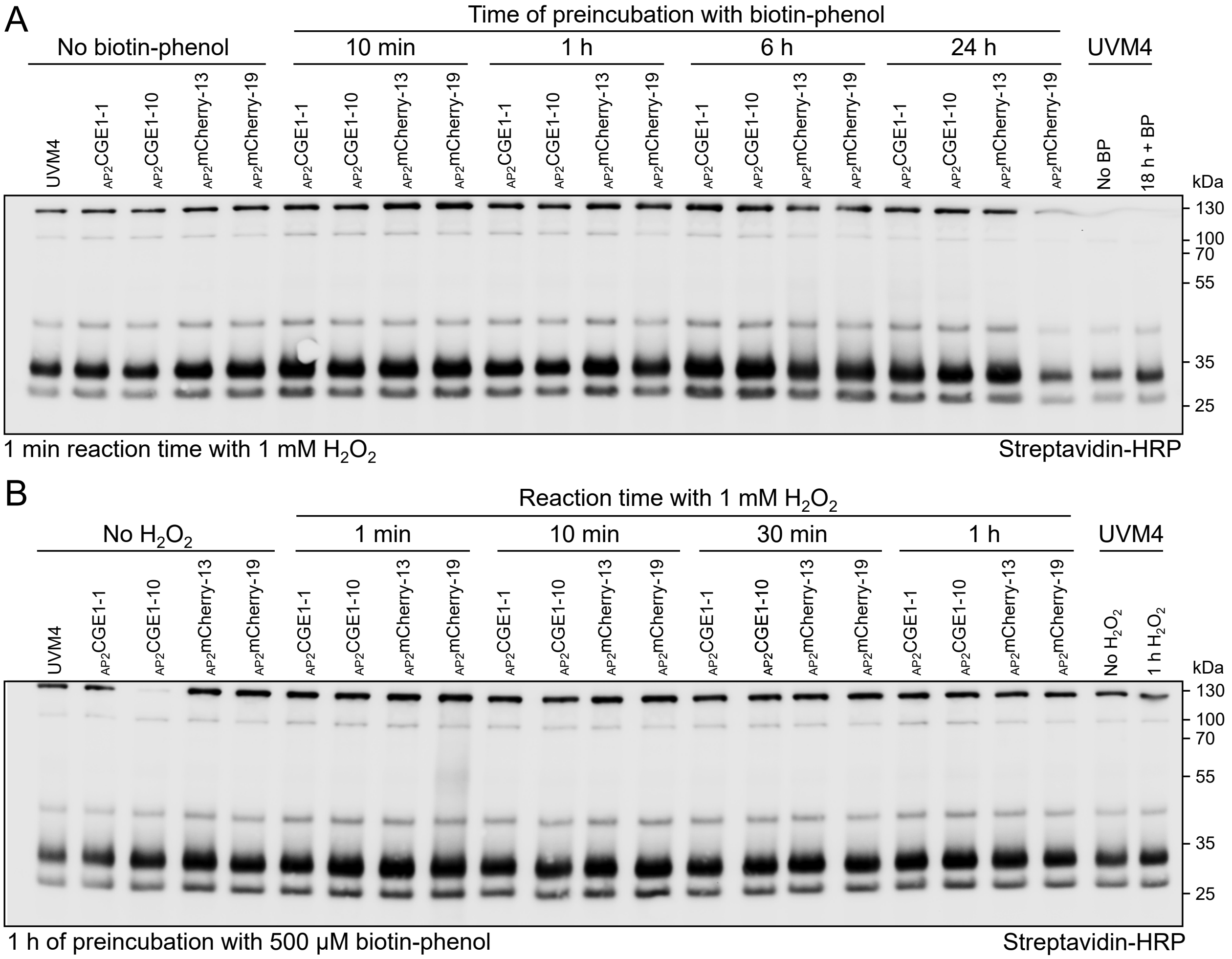

Supplemental Figure S3. Dependency of APEX2 activity on biotin-phenol preincubation time and labeling reaction time.

Mid-log phase *Chlamydomonas* cultures were supplemented with 500 µM biotin-phenol dissolved in DMSO and 1 mM H_2O_2. *In vivo* APEX2 activity was analyzed via immunoblotting on total protein extracts corresponding to 1 µg chlorophyll using streptavidin-HRP for the detection of biotinylated proteins.

(A) Varying preincubation time. *Chlamydomonas* cells were supplemented with 500 µM biotin-phenol for the indicated time at 22°C and the labeling reaction was allowed to proceed for 1 min after the addition of 1 mM H_2O_2.

(B) Varying labeling time. *Chlamydomonas* cells were supplemented with 500 µM biotin-phenol for 1 h at 22°C and the labeling reaction was allowed to proceed for the indicated time periods after the addition of 1 mM H_2O_2. Controls were performed with either biotin-phenol or H_2O_2 omitted. The UVM4 recipient strain was used as the peroxidase-omitted control.

Supports Figure 1.

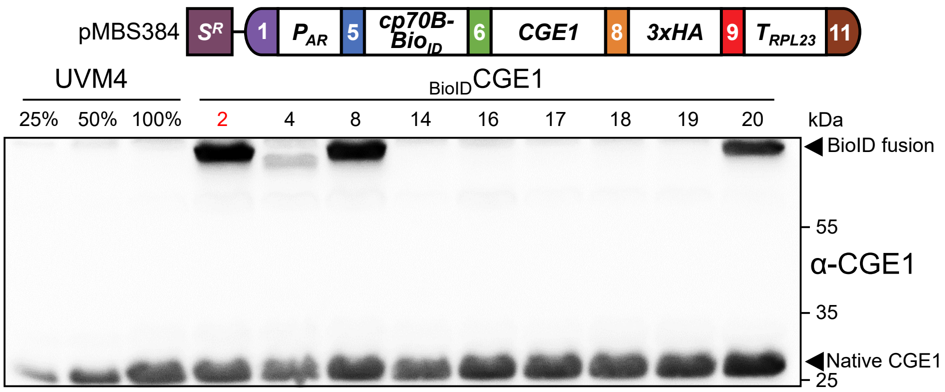

Supplemental Figure S4. Screening for transformants accumulating CGE1 N-terminally fused to BioID.

Level 2 construct pMBS384 conferring resistance to spectinomycin (S^R) and driving the production of CGE1 fused N-terminally to BioID ($_{BioID}$CGE1). The analysis of transformants generated with the constructs was done as described in Supplemental Figure S2.

Supports Figure 2.

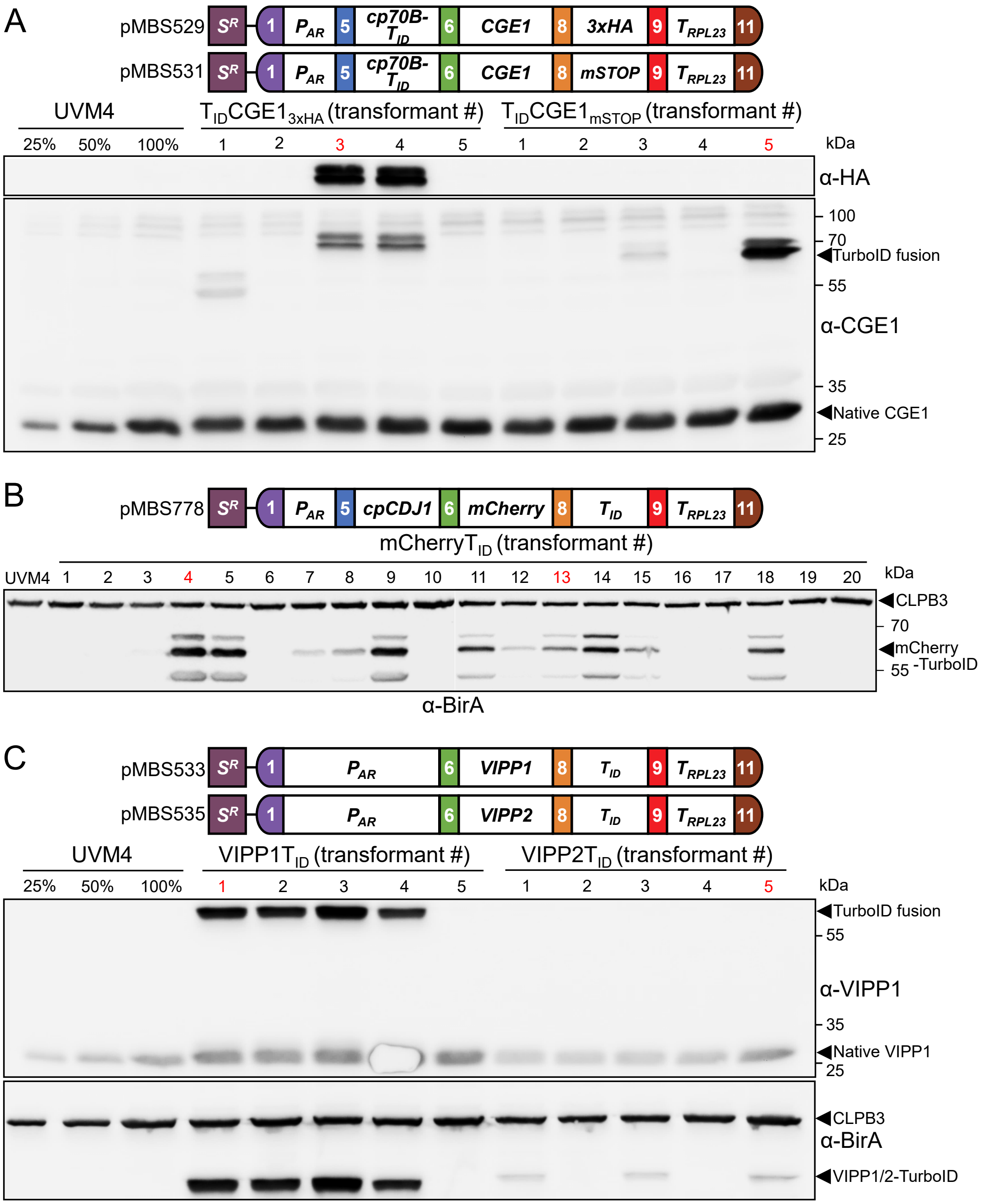

Supplemental Figure S5. Screening for transformants accumulating TurboID fusions to VIPP1, VIPP2, CGE1, and mCherry.

(A-C) Level 2 constructs for the production of CGE1, VIPP1, VIPP2, and mCherry fused to TurboID (T_{ID}). The analysis of transformants generated with the constructs was done as described in Supplemental Figure S2.

Supports Figure 2.

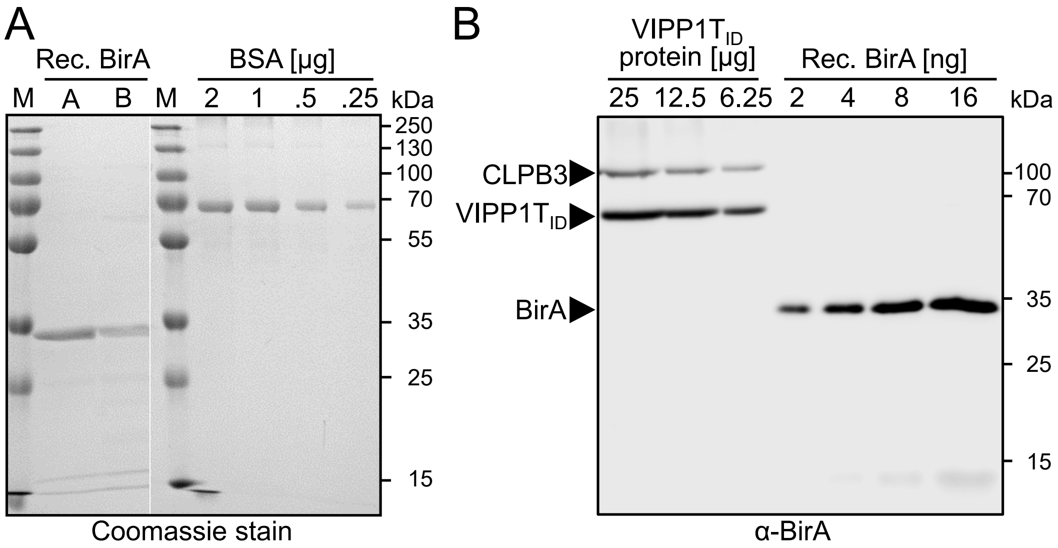

Supplemental Figure S6. Production of recombinant BirA and characterization of the antiserum raised against it.

(A) *E. coli* BirA was expressed with an N-terminal hexa-histidine tag in *E. coli* and purified by cobalt-nitrilotriacetic acid affinity chromatography followed by gel filtration on an Enrich SEC650 column. 2 µl of protein resulting from two independent preparations (A and B) were analyzed next to a dilution series of BSA on a 12% SDS-polyacrylamide gel and stained with Coomassie blue.

(B) 2 to 16 ng of recombinant BirA were separated by SDS-PAGE next to 6.25 to 25 µg of whole-cell proteins from a VIPP1T$_{ID}$ transformant and immunodetected with the antiserum raised against BirA. The antiserum detects recombinant BirA, the VIPP1T$_{ID}$ fusion protein, and cross-reacts with CLPB3 from *Chlamydomonas* chloroplasts.

Supports Figure 2.

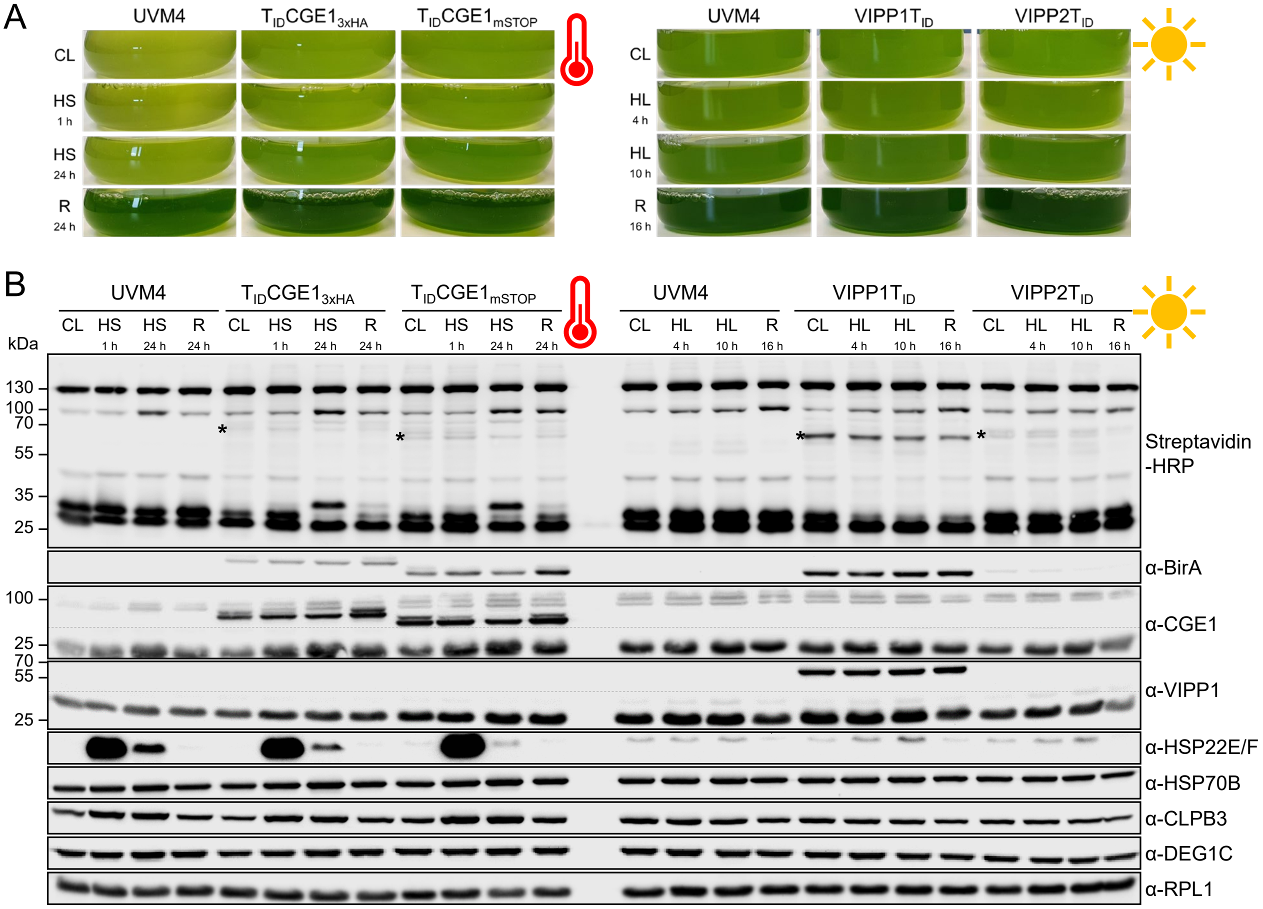

Supplemental Figure S7. Impact of TurboID mediated biotinylation on cell fitness.
Chlamydomonas cultures of the UVM4 recipient strain and transformants producing T_{ID}CGE1-3xHA, T_{ID}CGE1-mSTOP and VIPP1/2-T_{ID} were grown to mid-log phase at 22°C (CL). Cultures were then either exposed to 40°C for 24 h (HS, left) or to high light of 1000 µE m^{-2} s^{-1} for 10 h (HL, right) and were allowed to recover for 16-24 h at 22°C (R).
(A) Pictures of the cultures at the indicated time points during the stress treatments.
(B) Total cell protein extracts corresponding to 2 µg chlorophyll for each sample were separated by SDS-PAGE and analyzed by immunoblotting using streptavidin-HRP to detect biotinylated proteins, or antibodies to detect specifically the proteins indicated on the right. The asterisk points to the positions of self-biotinylated T_{ID} fusion proteins. Control samples were taken before the respective treatment (CL). The UVM4 strain serves as the ligase-omitted control.
Supports Figure 2.

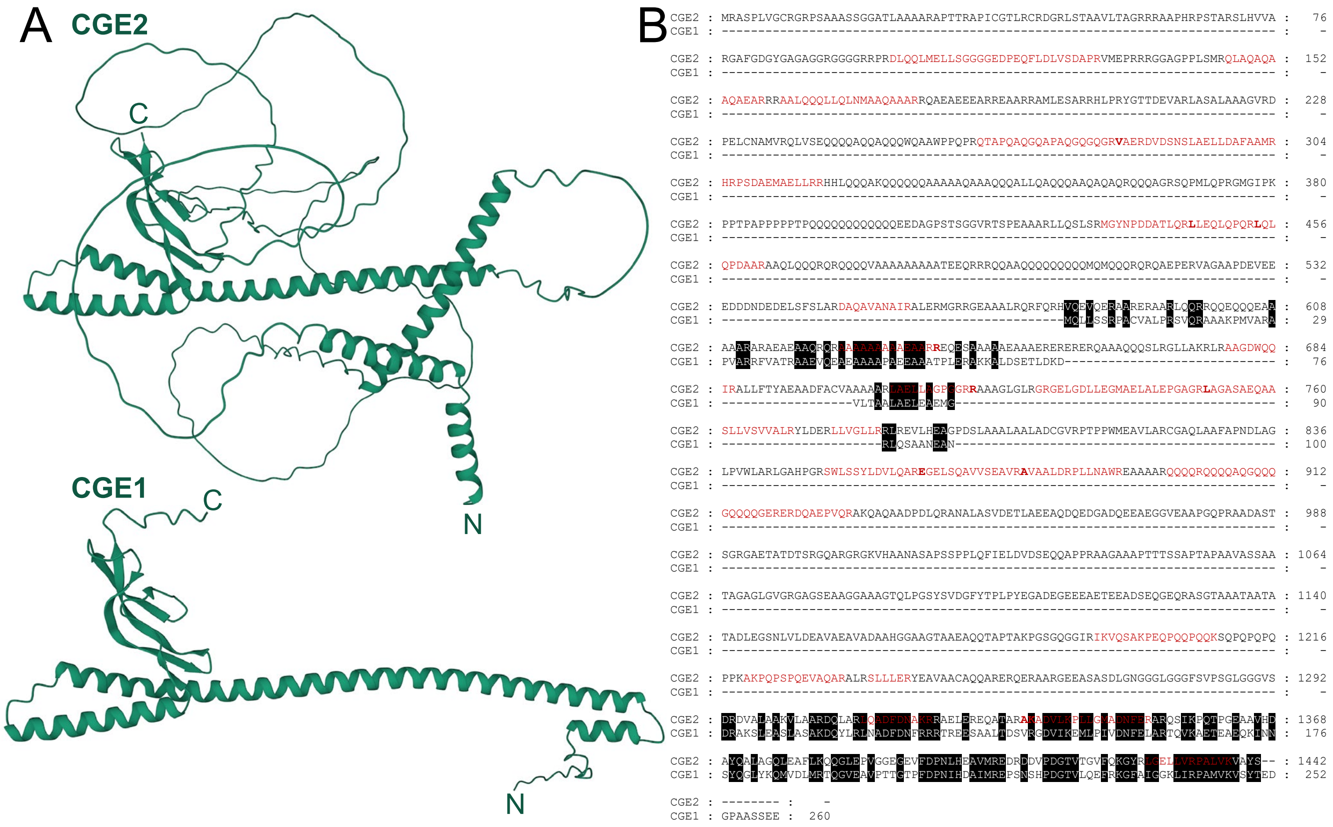

Supplemental Figure S8. Comparison of structures and sequences of CGE2 and CGE1.
(A) Alpha-fold structures of CGE2 and CGE1 (Jumper et al., 2021).
(B) Alignment of amino acid sequences of *Chlamydomonas* CGE2 and CGE1. The alignment was conducted with CLUSTALW and displayed by GeneDoc. Sequences in red indicate the 29 tryptic peptides identified for CGE2 in the TurboID-CGE1 proxiome (bold letters indicate the start of a new peptide if they are adjacent or additional sequences in missed cleavages).
Supports Figure 3.

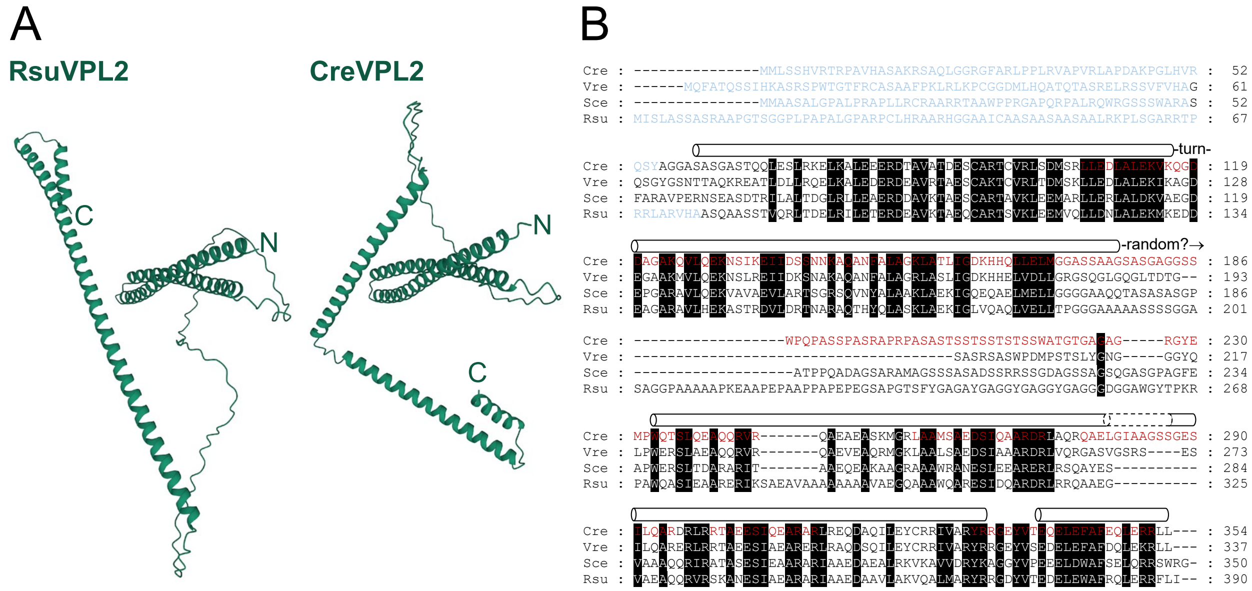

Supplemental Figure S9. Structures and alignments of putative VPL2 orthologs.
(A) Alpha-fold structures of VPL2 from *Raphidocelis subcapitata* (Rsu) and *Chlamydomonas reinhardtii* (Cre) (Jumper et al., 2021).
(B) Alignment of amino acid sequences of VPL2 from *Chlamydomonas reinhardtii* (Cre, Cre07.g333150), *Volvox reticuliferus* (Vre, GIL75541), *Scenedesmus sp.* (Sce, KAF8054895.1), and *Raphidocelis subcapitata* (Rsu, GBF91661). The alignment was conducted with CLUSTALW and displayed by GeneDoc. Chloroplast transit peptides predicted by TargetP (Almagro Armenteros et al., 2019) are shown in blue letters, sequences covered by overall 31 identified peptides in red letters. Alpha helices predicted by alpha-fold are indicated by rods.
Supports Figure 4.

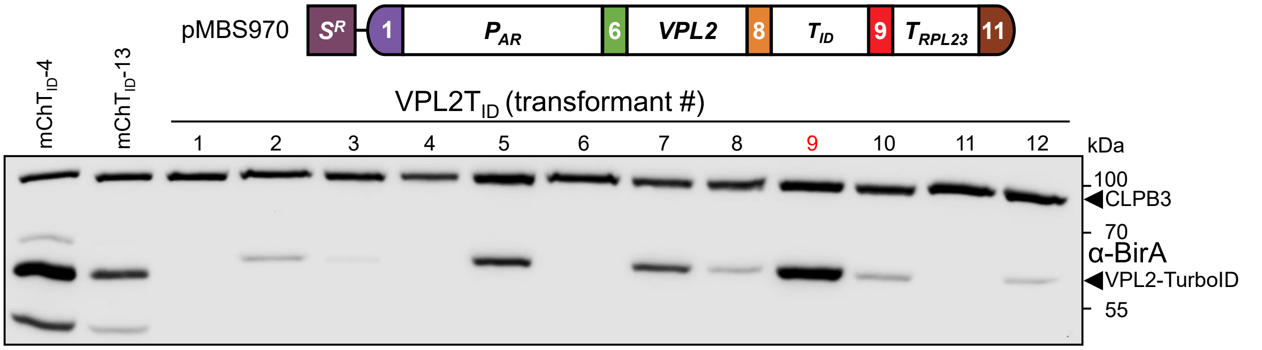

Supplemental Figure S10. Screening of transformants accumulating VPL2 with a C-terminal TurboID fusion.
Level 2 construct for the production of VPL2 fused C-terminally to TurboID (T_{ID}). The analysis of transformants generated with the construct was done as described in Supplemental Figure S2. mCherryT_{ID} lines served as positive control.
Supports Figure 5.

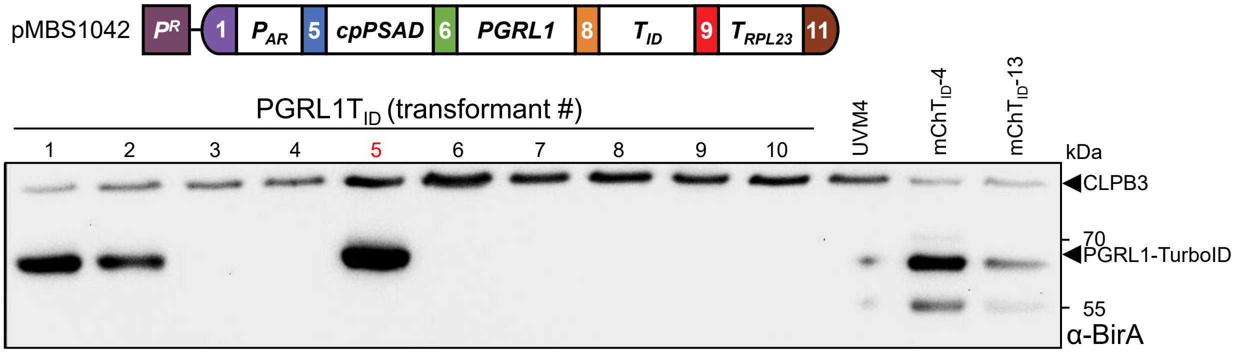

Supplemental Figure S11. Screening of transformants accumulating PGRL1 with a C-terminal TurboID fusion.

Level 2 construct for the production of PGRL1 fused C-terminally to TurboID (T_{ID}). The analysis of transformants generated with the construct was done as described in Supplemental Figure S2. mCherryT_{ID} lines served as positive control.

Supports Figure 5.

Supplemental Table S1. List of proteins in the VIPP1/2 proxiomes and upregulation of their encoding genes by chloroplast stresses in previous studies.

Gene identifier	Protein name	Functional annotation	Loc[a] T/P	Fold enrichment with VIPP1 CL/H_2O_2[b]	Fold enrichment with VIPP2 CL/H_2O_2[b]	TMH[c]	ClpP[d]	Cerulenin[e]	Ni^{2+} Ions[f]	H_2O_2[g]
Cre14.g617400	HSP22F	Molecular chaperone	cp	-	-	0	121	96	27	8.8
Cre12.g498500	DEG1C	Stromal protease	cp	-	-	0	81	36	11	3.2
Cre13.g583550	VIPP1	Thylakoid biogenesis/<br>Cp membrane maintenance	cp	**2930/921[s1]**<br>**2587/3172[s2]**<br>**140/148[s3]**	15.2/-[s1]<br>-/38.5[s2]<br>4.2/9.5[s3]	0	5.6	11	-	-
Cre11.g468050	VIPP2	Cp membrane maintenance	cp	-/212[s2]<br>-<br>-/51[s3]	**1318/724[s1]**<br>**1288/1616[s2]**<br>**1052/3246[s3]**	0	44	92	20	4.7
Cre02.g090850	CLPB3	Molecular chaperone	cp	-/10.1[s3]	-	0	9.4	10	2.3	5.1
Cre06.g250100	HSP70B	Molecular chaperone	cp	9.5/-[s2]<br>10/26[s3]	-	0	3.4	12.5	-	-
Cre09.g393200	HSP70C	Molecular chaperone	mt	12.7/26.4[s3]	-	0	2	2.5	-	2
Cre07.g316050	CDJ2	HSP70B co-chaperone<br>(VIPP1 assembly state)	cp	7.3/34.5[s3]	9.0/22[s3]	0	-	3	-	-
Cre03.g149250	VPL1/<br>ICL2	PEP carboxylase family	cp/cp	29/35[s1]<br>312/645[s3]	-<br>19/201[s3]	0	13.2	21	3.9	2.7
Cre07.g333150	VPL2	Unknown function	cp	281/283[s1]<br>342.6/344[s2]<br>1018/1163[s3]	19.5/15.6[s1]<br>-/19.4[s2]<br>70/646[s3]	0	12	27	4.1	2.2
Cre07.g338350	VPL3/<br>CPLD50	Fe-S cluster<br>biosynthesis family	mt/cp	-/60.3[s2]<br>-/14.8[s3]	-/21.7[s2]<br>-/24.9[s3]	4	6.3	5.7	2.2	3.5
Cre01.g045600	VPL4/<br>SRR12	S/T protein kinase	mt/cp	-/21.4[s3]	-/15.6[s3]	2	3.6	7.8	-	2.6
Cre16.g687200	VPL5	Unknown function	mt/cp	-/10.3[s3]	-	2	-	3	-	-
Cre03.g179800	VPL6/<br>LCI24	Low-CO_2-inducible protein,<br>unknown function	mt/cp	17.2/-[s2]<br>-/11.4[s3]	-<br>-	1	-	2	-	-
Cre13.g570350	VPL7/<br>AKC4	ABC1 atypical kinase	mt/cp	11.3/5.3[s3]	10.9/-[s3]	0	2.4	5.2	-	-
Cre07.g333350	VPL8	Unknown function	cp/cp	-/34[s3]	-	0	9	12	-	2.7
Cre13.g570400	VPL9	Chlorophyllase	cp/cp	6.4/-[s3]	-	0	4.6	6.6	-	-
Cre12.g547250	VPL10	Unknown function	mt/cp	-/33[s3]	-	0	2.7	3.2	-	-
Cre13.g604150	VPL11/<br>ABCB4	Half-size ABC transporter	mt/mt	-	6.2/-[s3]	6	5.4	3.8	-	-
Cre01.g050950	CHLP1	Geranyl-geranyl reductase	cp/cp	4.0/-[s3]	3.0/-[s3]	0	-	2.3	-	-
Cre03.g184550	LPA3/<br>CPLD28	PS II assembly factor	cp	-/11.5[s3]	-	0	-	2.6	-	-

Supplemental Table S1. Continued.

Cre05.g247450	RDP5/ CGL56	Rhodanese-like protein, Calcium sensing receptor-like	mt/cp	-/13.5[s1] 11.7/-[s2] 10.5/-[s3]	14/26.4[s1] - 15/7.8[s3]	0	-	-	-	-
Cre07.g340200	TEF3/ PGRL1	Proton-gradient regulation like 1 (regulation of CEF)	cp	- -	6.1/-[s1] 10/-[s3]	2	-	-	-	-
Cre05.g245158	GET3B	Tail-anchored protein targeting to thylakoid membranes	cp	23/70[s3]	-/15.6[s3]	0	-	-	-	-
Cre01.g004000		DUF1825 (cyanobacteria)	cy/cy	-/33.5[s3]	-	0	-	-	-	-
Cre10.g446100	TRXy	Chloroplastic thioredoxin y	cp	-/7.4[s3]	-	0	-	-	-	-
Cre02.g142800	TRXz	Chloroplastic thioredoxin z	cp	-/5.3[s3]	-	0	-	-	-	-
Cre13.g562750	CGLD38	DUF4336	mt/cp	-/5.8[s3]	7.1/-[s3]	0	-	-	-	-
Cre18.g748397	CGL143	Unknown function	mt/mt	-/32[s3]	-	0	-	-	-	-
Cre06.g280650	CGL59/ Y3IP1	PS I biogenesis factor	cp	7.2/-[s3]	-	1	-	-	-	-
Cre07.g326050	KIL8	Kinesin-like motor protein	cp/mt	19.5/-[s3]	-	0	-	-	-	-
Cre06.g269050	TIC62	Membrane anchor for FNR	cp	5.0/3.1[s1]	4.8/-[s1]	0	-	-	-	-
Cre12.g519100	ACC1	Acetyl-CoA carboxylase	mt/cp	-/3.9[s1]	-	0	-	-	-	-
Cre10.g434750	AAI1	Acetohydroxy acid isomer-reductase (branched chain amino acid synthesis)	mt/cp	-/10.9[s1]	-	0	-	-	-	-
Cre09.g386735	DLA1	Dihydrolipoamide acety-ltransferase (E2 subunit of mitochondrial PDC)	mt/mt	-	-/10.8[s3]	0	-	-	-	-
Cre01.g049600	CGLD22/ CGL160	Cp ATPase assembly	cp	-	7.9/8.1[s1]	4	-	-	-	-
Cre10.g430150	LPA1	PS II assembly factor	cp	-	4.4/-[s3]	2	-	-	-	-
Cre04.g220200	KEA1	K^+ efflux antiporter	cp	5.3/7.1[s1]	-	14	-	-	-	-
Cre06.g278269		Unknown function	-/sp	14.6/-[s3]	-	0	-	-	-	-
Cre06.g259100		Unknown function	mt/cp	6.2/-[s1]	-	0	-	-	-	-
Cre07.g325736	PAP	Plastid lipid-associated protein/fibrillin	mt/cp	4.2/4.6[s1]	4.0/-[s1]	0	-	-	-	-
Cre12.g512300	LOX	Putative lipoxygenase	mt/mt	9.1/6.2[s1] -/6.9[s2]	- -	0	-	-	-	-
Cre09.g416850		Unknown function (contains rhodanese-like domain)	cp/mt	-	-/7.6[s3]	0	-	-	-	-

[a] Localization as predicted by TargetP (T) (Almagro Armenteros et al., 2019) and Predalgo (P) (Tardif et al., 2012). cp – chloroplast; mt – mitochondria; cy – cytosol; sp – secretory pathway. A single localization indicates that it is based on experimental evidence.
[b] enrichment in experimental setup s1, s2 or s3
[c] transmembrane helices (TMH) predicted by DeepTMHMM (Hallgren et al., 2022)
[d] 43 h after addition of vitamins to induce the depletion of ClpP (Ramundo et al., 2014)
[e] 4 h after cerulenin addition (Heredia-Martínez et al., 2018)
[f] 6 h after the addition of 50 µM $NiCl_2$ (Blaby-Haas et al., 2016)
[g] 1 h after addition of 1 mM H_2O_2 (Blaby et al., 2015)
[d-g] Values are fold upregulation as determined by RNA-seq in the indicated studies. For comparison, values for cpUPR marker genes *HSP22F* and *DEG1C* were added.

Supplemental Table S2. Primers used for cloning.

Primer	Sequence	Target, product size
CGE1g-1	5’-AA**GAAGAC**AAAATGGTACAGGTCTGCAAATTGCAC-3’	*CGE1*, 208 bp
CGE1g-2	5’-TT**GAAGAC**TTCGTCTCGCTGTCCAGAGCCTTC-3’	
CGE1g-3	5’-AA**GAAGAC**GAGACGCTGGACAAGGATGTGCTG-3’	*CGE1*, 1776 bp
CGE1g-4	5’-TT**GAAGAC**GCCTCCGTCTCCGCCTTGACCTG-3’	
CGE1g-5	5’-AA**GAAGAC**ACGGAGGCGGAGCAGAAGATCAA-3’	*CGE1*, 739 bp
CGE1g-6	5’-TT**GAAGAC**CCATCTTCTGCCAGTCCCATGCA-3’	
CGE1g-7	5’-AA**GAAGAC**GAAGATGGACCGGGGCTGTTGGGGT-3’	*CGE1*, 617 bp
CGE1g-8	5’-TT**GAAGAC**TTCGAACCCTCCTCAGAGCTAGCCGCAG-3’	
CTP-BirA-1	5’-TT**GAAGAC**AACCATGCCGGTTCAGCAGATGAC-3’	*HSP70B*, 249 bp
CTP-APEX2-2	5’-TT**GAAGAC**AAAGTCCTGAAGGAACAATTCAAATGTG-3’	
APEX2-C-for	5’-TTAA**GAAGAC**TTTTCGGACTACAAGGACGACGACGAC-3’	*APEX2*, 986 bp
APEX2-C-rev	5’-AAAA**GAAGAC**AAAAGCTAGTCCAGGGTCAGGCGCTCCA-3’	
CTP-BirA-1	5’-TT**GAAGAC**AACCATGCCGGTTCAGCAGATGAC-3’	*HSP70B,* 164 bp
CTP-BirA-2	5’-TT**GAAGAC**TTAAGACCGCTTTCGCACCTTCTC-3’	
CTP-BirA-3	5’-TT**GAAGAC**AGTCTTAACCCACCGACGCCCGTC-3’	*70B, BirA,* 183 bp
CTP-BirA-4	5’-TT**GAAGAC**AACGTCTCGCCCAGCTGCTCGCC-3’	
CTP-BirA-5	5’-TT**GAAGAC**GAGACGCTGGGCATGAGCCGCGCCGCC-3’	*BirA, 70B* 1177 bp
CTP-BirA-6	5’-TT**GAAGAC**AGCATTGAGCCACCGCCGCCCTTCTCG-3’	
CTP-BirA-1	5’-TT**GAAGAC**AACCATGCCGGTTCAGCAGATGAC-3’	*TurboID*, 245 bp
CTP-BirA-#2b	5’-TT**GAAGAC**AACTGAAGGAACAATTCAAATGTGA-3’	
BirA-Bam	5’-aaaaggatccAAGGATAACACCGTGCCACTG-3’	*E. coli* BirA, 981 bp
BirA-Hind	5’-ttttaagcTTATTTTTCTGCACTACGCAGG-3’	

BbsI recognition sites are shown in bold and produced overhangs are underlined.

Supplemental Table S3. MoClo constructs employed and generated.

Name	Resistance		A1-B2				B3-B4		B5		B6-C1		Level 1	Level 2
$_{APEX2}$CGE1	S^R	pCM1-01	P_{AR}	pCM0-15	APEX2	pMBS454	CGE1	pMBS375	3xHA	pCM0-100	T_{RPL23}	pCM0-119	pMBS500	pMBS501
$_{APEX2}$mCherry	S^R	pCM1-01	P_{AR}	pCM0-15	APEX2	pMBS454	mCherry	pCM0-067	3xHA	pCM0-100	T_{RPL23}	pCM0-119	pMBS779	pMBS780
Bio$_{ID}$CGE1	S^R	pCM1-01	P_{AR}	pCM0-15	B_{ID}	pMBS197	CGE1	pMBS375	3xHA	pCM0-100	T_{RPL23}	pCM0-119	pMBS383	pMBS384
T_{ID}CGE1$_{3xHA}$	S^R	pCM1-01	P_{AR}	pCM0-15	T_{ID}	pMBS515	CGE1	pMBS375	3xHA	pCM0-100	T_{RPL23}	pCM0-119	pMBS528	pMBS529
T_{ID}CGE1$_{mSTOP}$	S^R	pCM1-01	P_{AR}	pCM0-15	T_{ID}	pMBS515	CGE1	pMBS375	mSTOP	pCM0-101	T_{RPL23}	pCM0-119	pMBS530	pMBS531
mCherryT_{ID}	S^R	pCM1-01	P_{AR}	pCM0-15	CDJ1	pMBS640	mCherry	pCM0-067	T_{ID}	pMBS512	T_{RPL23}	pCM0-119	pMBS777	pMBS778
PGRL1T_{ID}	P^R	pCM1-27	P_{AR}	pCM0-15	PSAD	pCM0-52	PGRL1	pMBS1045	T_{ID}	pMBS512	T_{RPL23}	pCM0-119	-	pMBS1042
VIPP1T_{ID}	S^R	pCM1-01	P_{AR}			pCM0-20	VIPP1	pMBS478	T_{ID}	pMBS512	T_{RPL23}	pCM0-119	pMBS532	pMBS533
VIPP2T_{ID}	S^R	pCM1-01	P_{AR}			pCM0-20	VIPP2	pMBS277	T_{ID}	pMBS512	T_{RPL23}	pCM0-119	pMBS534	pMBS535
VPL2T_{ID}	S^R	pCM1-01	P_{AR}			pCM0-20	VPL2	pMBS969	T_{ID}	pMBS512	T_{RPL23}	pCM0-119	-	pMBS970

pCM plasmids were constructed by Crozet et al. (2018), pMBS277 by Theis et al. (2020), pMBS478 by Gupta et al. (2021) and pMBS640 by Niemeyer et al. (2021). All other plasmids were made in this study. The positions in the MoClo constructs are according to Patron et al. (2015).

Supplemental Table S4. Predicted molecular masses of fusion proteins.

Level 2 plasmid	Protein	Precursor (Da)	Mature (Da)
pMBS501	cp70B-APEX2-CGE1-3xHA	61451.82	57390.02
pMBS780	cp70B-APEX2-mCherry-3xHA	64274.35	60212.55
pMBS384	cp70B-BioID-CGE1-3xHA	67285.40	63223.60
pMBS529	cp70B-TurboID-CGE1-3xHA	67879.23	63817.43
pMBS531	cp70B-TurboID-CGE1	64194.39	60132.59
pMBS778	cpCDJ1-mCherry-TurboID	67515.36	62853.97
pMBS533	VIPP1-TurboID	67500.50	63523.84
pMBS535	VIPP2-TurboID	77811.92	65614.18
pMBS970	VPL2-TurboID	73857.02	67905.01
pMBS1042	PGRL1-TurboID	71334.53	67701.17

References

Almagro Armenteros JJ, Salvatore M, Emanuelsson O, Winther O, von Heijne G, Elofsson A, Nielsen H (2019) Detecting sequence signals in targeting peptides using deep learning. Life Sci Alliance **2**

Blaby-Haas CE, Castruita M, Fitz-Gibbon ST, Kropat J, Merchant SS (2016) Ni induces the CRR1-dependent regulon revealing overlap and distinction between hypoxia and Cu deficiency responses in *Chlamydomonas reinhardtii*. Metallomics **8:** 679-691

Blaby IK, Blaby-Haas CE, Perez-Perez ME, Schmollinger S, Fitz-Gibbon S, Lemaire SD, Merchant SS (2015) Genome-wide analysis on *Chlamydomonas reinhardtii* reveals the impact of hydrogen peroxide on protein stress responses and overlap with other stress transcriptomes. Plant J **84:** 974-988

Crozet P, Navarro FJ, Willmund F, Mehrshahi P, Bakowski K, Lauersen KJ, Perez-Perez ME, Auroy P, Gorchs Rovira A, Sauret-Gueto S, Niemeyer J, Spaniol B, Theis J, Trosch R, Westrich LD, Vavitsas K, Baier T, Hubner W, de Carpentier F, Cassarini M, Danon A, Henri J, Marchand CH, de Mia M, Sarkissian K, Baulcombe DC, Peltier G, Crespo JL, Kruse O, Jensen PE, Schroda M, Smith AG, Lemaire SD (2018) Birth of a photosynthetic chassis: a MoClo toolkit enabling Synthetic Biology in the microalga *Chlamydomonas reinhardtii*. ACS Synth Biol **7:** 2074-2086

Gupta TK, Klumpe S, Gries K, Heinz S, Wietrzynski W, Ohnishi N, Niemeyer J, Spaniol B, Schaffer M, Rast A, Ostermeier M, Strauss M, Plitzko JM, Baumeister W, Rudack T, Sakamoto W, Nickelsen J, Schuller JM, Schroda M, Engel BD (2021) Structural basis for VIPP1 oligomerization and maintenance of thylakoid membrane integrity. Cell **184:** 3643-3659 e3623

Hallgren J, Tsirigos KD, Pedersen MD, Almagro Armenteros JJ, Marcatili P, Nielsen H, Krogh A, Winther O (2022) DeepTMHMM predicts alpha and beta transmembrane proteins using deep neural networks. bioRxiv**:** 2022.2004.2008.487609

Heredia-Martínez LG, Andrés-Garrido A, Martínez-Force E, Pérez-Pérez ME, Crespo JL (2018) Chloroplast damage induced by the inhibition of fatty acid synthesis triggers autophagy in *Chlamydomonas*. Plant Physiol **178:** 1112-1129

Jumper J, Evans R, Pritzel A, Green T, Figurnov M, Ronneberger O, Tunyasuvunakool K, Bates R, Žídek A, Potapenko A, Bridgland A, Meyer C, Kohl SAA, Ballard AJ, Cowie A, Romera-Paredes B, Nikolov S, Jain R, Adler J, Back T, Petersen S, Reiman D, Clancy E, Zielinski M, Steinegger M, Pacholska M, Berghammer T, Bodenstein S, Silver D, Vinyals O, Senior AW, Kavukcuoglu K, Kohli P, Hassabis D (2021) Highly accurate protein structure prediction with AlphaFold. Nature **596:** 583-589

Niemeyer J, Scheuring D, Oestreicher J, Morgan B, Schroda M (2021) Real-time monitoring of subcellular H_2O_2 distribution in *Chlamydomonas reinhardtii*. Plant Cell **33:** 2935-2949

Patron NJ, Orzaez D, Marillonnet S, Warzecha H, Matthewman C, Youles M, Raitskin O, Leveau A, Farre G, Rogers C, Smith A, Hibberd J, Webb AA, Locke J, Schornack S, Ajioka J, Baulcombe DC, Zipfel C, Kamoun S, Jones JD, Kuhn H, Robatzek S, Van Esse HP, Sanders D, Oldroyd G, Martin C, Field R, O'Connor S, Fox S, Wulff B, Miller B, Breakspear A, Radhakrishnan G, Delaux PM, Loque D, Granell A, Tissier A, Shih P, Brutnell TP, Quick WP, Rischer H, Fraser PD, Aharoni A, Raines C, South PF, Ane JM, Hamberger BR, Langdale J, Stougaard J, Bouwmeester H, Udvardi M, Murray JA, Ntoukakis V, Schafer P, Denby K, Edwards KJ, Osbourn A, Haseloff J (2015) Standards for plant synthetic biology: a common syntax for exchange of DNA parts. New Phytol **208:** 13-19

Ramundo S, Casero D, Mühlhaus T, Hemme D, Sommer F, Crevecoeur M, Rahire M, Schroda M, Rusch J, Goodenough U, Pellegrini M, Perez-Perez ME, Crespo JL, Schaad O, Civic N, Rochaix JD (2014) Conditional depletion of the *Chlamydomonas* chloroplast ClpP protease activates nuclear genes involved in autophagy and plastid protein quality control. Plant Cell **26:** 2201-2222

Tardif M, Atteia A, Specht M, Cogne G, Rolland N, Brugière S, Hippler M, Ferro M, Bruley C, Peltier G, Vallon O, Cournac L (2012) PredAlgo: a new subcellular localization prediction tool dedicated to green algae. Mol Biol Evol **29:** 3625-3639

Theis J, Niemeyer J, Schmollinger S, Ries F, Rutgers M, Gupta TK, Sommer F, Muranaka LS, Venn B, Schulz-Raffelt M, Willmund F, Engel BD, Schroda M (2020) VIPP2 interacts with VIPP1 and HSP22E/F at chloroplast membranes and modulates a retrograde signal for HSP22E/F gene expression. Plant Cell Environ **43:** 1212-1229
